# Super-resolved analysis of colocalization between replication and transcription along the cell cycle in a model of oncogene activation

**DOI:** 10.1038/s42003-024-06972-2

**Published:** 2024-10-04

**Authors:** Anna Provvidenza Privitera, Silvia Scalisi, Greta Paternò, Elena Cerutti, Morgana D’Amico, Pier Giuseppe Pelicci, Mario Faretta, Gaetano Ivan Dellino, Alberto Diaspro, Luca Lanzanò

**Affiliations:** 1 Department of Physics and Astronomy “Ettore Majorana”, University of Catania, Catania, Italy; 2 Nanoscopy and NIC@IIT, CHT Erzelli, Istituto Italiano di Tecnologia, Genoa, Italy; 3 European Institute of Oncology IRCCS, Milan, Italy; 4 Department of Oncology and Hemato-Oncology, University of Milan, Milan, Italy; 5 DIFILAB, Department of Physics, University of Genoa, Genoa, Italy

**Keywords:** Nanoscale biophysics, Super-resolution microscopy, Oncogenes

## Abstract

To understand how oncogenes affect genome organization, it is essential to visualize fundamental processes such as DNA replication and transcription at high resolution in intact cells. At the same time, it is important to determine the progression of the cell along the cell cycle, as cell cycle regulation is crucial for the control of cell proliferation and oncogenesis. Here, we present a super-resolution imaging-based method to analyze single cell nuclei sorted according to specific phases of the cell cycle. The sorting is based on the evaluation of the number and the intensity of pixels in the replication foci image and the colocalization analysis is based on image cross-correlation spectroscopy (ICCS). We evaluate the colocalization between replication and transcription, at different cell cycle phases, in a model of PML-RARα oncogene activation. We find that colocalization between replication and transcription is higher in cells in early S phase compared to cells in middle and late S phase. When we turn on the PML-RARα oncogene, this colocalization pattern is preserved but we detect an increase of colocalization between replication and transcription in the early S phase which points to an effect of the PML-RARα oncogene on the coordination between replication and transcription.

## Abstract (web summary)

A super-resolution imaging method for cell cycle sorting reveals that the PML-RARα oncogene induces an increase in colocalization between replication and transcription in early S phase.

## Introduction

Several forms of cancer are triggered by the activation of specific oncogenes^1^. As a representative example, acute promyelocytic leukemia (APL) is a disease characterized by the accumulation of malignant promyelocytes blasts in the bone marrow^2,3^. The genetic cause of APL phenotype is due to a balanced reciprocal chromosomal translocation t(15;17) which occurs in 100% of APL cases and produces an anomalous fusion protein known as Promyelocytic leukemia protein (PML) - Retinoic Acid Receptor Alpha (RARα) (PML-RARα)^4,5^. The U937-PR9 cell line is a suitable model to investigate APL in vitro because it has been engineered by introducing an inducible PML-RARα oncogene^6^. The original cell line, U937, was obtained from malignant cells of generalized histiocytic lymphoma^7^. The peculiarity of U937-PR9 regards the possibility to induce expression of the PML-RARα oncogene by treating the cells with ZnSO_4_ for a certain time (usually 8 h and 24 h). Thus, U937-PR9 cells represent a suitable in vitro model for studying the impact of the PML-RARα oncogene on chromatin organization.

Recently, increasing interest has been focused on single cell imaging of cellular processes thanks to the technological improvements in the optical microscopy field. In particular, high resolution fluorescence microscopy techniques, such as confocal microscopy, have increased three-dimensional analysis capability, multicolor capability and number of samples observable in a certain time; all these improvements have allowed an automation of sample image analysis and an increase of statistical power^8–13^. The development of super-resolution microscopy has pushed the spatial resolution of fluorescence microscopy from the diffraction limit of 200 nm down to the nanometer scale, enabling the observation of finer details inside cells^13–15^.

In parallel with the advantages of confocal and super-resolution techniques, a growing interest was put on the study of chromatin topology and its complex spatial and temporal organization in the cell nucleus^13,16,17^. Under the microscope, one of the most evident levels of chromatin organization is the differentiation between euchromatin and heterochromatin. Euchromatin is rich in genes, it is not very condensed, and its replication occurs mainly in the so-called Early-S-Subphase of the cell cycle. In contraposition heterochromatin is highly compacted and mainly transcriptionally inert. Its replication occurs mainly in the Middle- and Late-S-Subphase of the cell cycle^18–20^. Super-resolution microscopy has shown that chromatin is organized at a scale between 10 nm and 200 nm^21,22^ and that this nanoscale organization is altered in cancer^17^.

Recently, we proposed a method to quantify oncogene-induced alterations in the organization of chromatin in single cell nuclei^23^. This method evaluates the spatial organization of functional sites in multiple cells by using an iterative algorithm based on image cross-correlation spectroscopy (ICCS)^24–26^. In particular, the ICCS implementation simplifies the colocalization analysis by skipping the pre-segmentation of the image into objects, making this approach suitable also when segmentation of images into objects is less accurate^23,27^. Application of the ICCS method to U937-PR9 cells revealed that, in response to activation of the PML-RARα oncogene, an increased fraction of transcription sites colocalized with PML/PML-RARα, following disruption of physiological PML bodies and the abnormal occurrence of a relatively large number of PML-RARα microspeckles. Unfortunately, in the approach of Cerutti et al. there is no information about the progression of the cells along the cell cycle^28^, information which is crucial to investigate the impact of oncogenes on the spatio-temporal coordination of nuclear functions^1^.

Here, we present a method to analyze single U937-PR9 cell nuclei labeled with a replication foci marker and sort the cells according to different subphases of S phase of the cell cycle. The method is based on the analysis of single optical sections acquired with confocal microscopy or super-resolved stimulated emission depletion (STED) microscopy^29^. As a replication foci marker, we use incorporation of 5-ethynyl-2′-deoxyuridine (EdU) coupled with click-chemistry fluorescent labeling. The sorting in our method is based only on the evaluation of the number and the intensity of pixels in a single optical section of the replication foci image, in contrast to standard methods based on the evaluation of the total content of EdU and DNA from 3D stacks^28,30^. Consequently, the definition of the early, middle, and late subphases in our work is slightly different compared to previously reported methods. More specifically, early and late correspond to shorter temporal windows at the begin and at the end of the S phase (replication foci patterns with low pixel density), respectively, whereas the middle subphase, as defined in our work, has a wider temporal span (replication foci patterns with high pixel density).

This approach enables us to measure the ICCS colocalization on cells sorted according to different cell cycle phases. As a validation, we monitor the colocalization between replication and transcription foci (identified by labeling RNA polymerase II). As expected, we find that colocalization between replication and transcription is higher in cells in early S phase compared to cells in middle and late S phase. This is in keeping with the fact that the more euchromatic regions of the genome are duplicated in the early S phase whereas the more heterochromatic regions are duplicated in the middle and late S phases, following a well-defined replication program. When we turn on the PML-RARα oncogene, we find a similar colocalization pattern, indicating that, at least in general, this program is preserved. Nevertheless, we detect an increase of colocalization between replication and transcription for cells in the early S phase (significance *p* = 0.012) which might be the indication of an effect of the PML-RARα oncogene on the coordination between replication and transcription.

## Results

### Automatic sorting of U937-PR9 cells according to the cell cycle phase

**Fig. 1:**
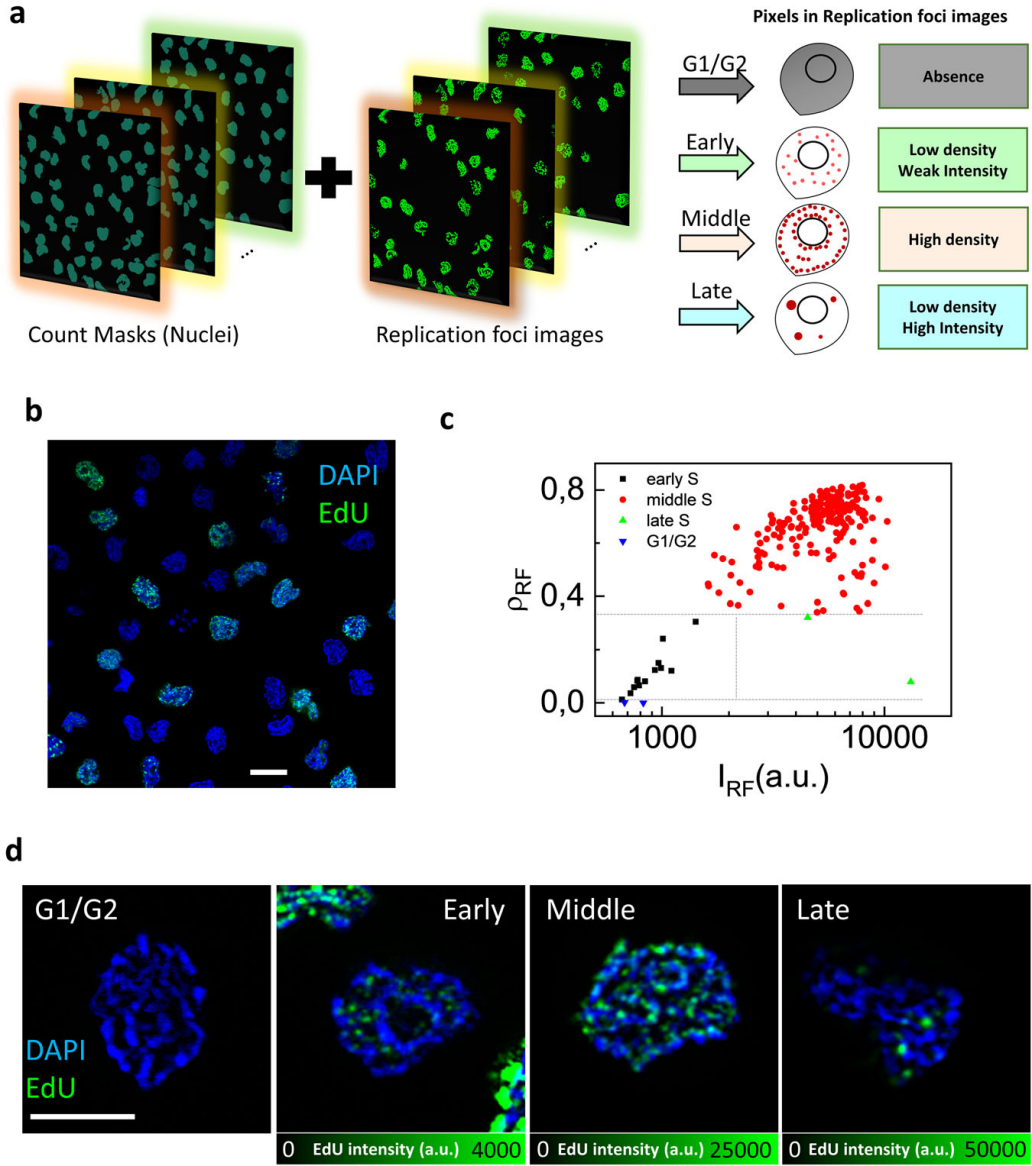
Method for sorting the cells according to progression along the cell cycle. **a** Schematic of the method for sorting the cells according to progression along the cell cycle: an algorithm processes multiple images and extracts the number and the average intensity of pixels in the replication foci image for each nucleus. The cells are then sorted into four groups (G1/G2, Early, Middle, Late), based on the values of density and intensity of pixels. **b** Representative confocal image of U937-PR9 cells labeled with DAPI (blue) and EdU-Alexa 488 (green). Scale bar 10 µm. **c** Scatter-plot of replication foci pixel density (ρ_RF_) versus intensity (*I*_RF_): the gray dashed lines indicate the thresholds for separation into the 4 groups. Each data point represents a single cell. Early S-subphase (black), Middle S-subphase (red), Late S-subphase (green), and G1/G2 phases (blue). Note that G1/G2 cells with *I*_RF_ = 0 are not visible in the plot. **d** Representative confocal images of EdU replication foci in U937-PR9 cells extracted from each group. Nuclear DNA is counterstained with DAPI. Scale bar 5 µm.

To sort cells according to the cell cycle phase, we labeled the cells with a DNA dye and a replication foci marker. The DNA dye was used to identify and segment the cell nuclei. The replication foci marker was used to identify the cell cycle phase^31^. The workflow of the method is schematically shown in Fig. 1a. The DNA images were used to identify the cell nuclei and convert them into objects in “Count Masks”. The replication foci images were used to (i) count the density of pixels in each nucleus (i.e. fraction of pixels of the nucleus showing replication foci signal) and (ii) measure the average intensity in each nucleus. To sort the cells we then used the following rationale: cells showing no replication foci were classified as cells in G1 or G2 phase (G1/G2); cells showing low density and weak intensity of replication foci pixels were classified as cells in Early-S-Subphase (Early); the cells showing high density of replication foci pixels were classified as cells in Middle-S-Subphase (Middle); cells showing low density and high intensity of replication foci pixels were classified as cells in Late-S-Subphase (Late). Nuclei of cells in mitosis were not included in the analysis. A potential pitfall in this scheme is represented by the very Late replicating cells entering G2, which tend to have low pixel density but not high pixel intensity and could be misclassified as Early. We assume that these cells can be recognized (and excluded) based on their larger-than-average nuclear size.

We first tested the algorithm on confocal images of U937-PR9 cells (Fig. 1b). Replication foci were labeled by using 5-ethynyl-2′-deoxyuridine (EdU) incorporation (25 min pulse labeling) followed by labeling with Alexa 488 azide, DNA was stained with DAPI. The analysis included 10 images with an image size of ~100 × 100 μm of the sample. The scatter plot in Fig. 1c shows the replication foci pixel density vs intensity distribution of the 408 cells analyzed. All cells showing no EdU signal (number of pixels below 10) were classified as G1/G2 (185 cells). All cells with a density above a given threshold ρ_thr_ were classified as Middle (207 cells). Cells with a density below the threshold ρ_thr_ and intensity below a threshold I_thr_ were classified as Early (13 cells). Cells with a density below the threshold ρ_thr_ and intensity above the threshold I_thr_ were classified as Late (2 cells). One cell was excluded because falling in the Early group but with nuclear size larger than the threshold. The thresholds were selected as described in the methods, visualizing the scatter plot (Supplementary Fig. S1) and the images of the cells at the boundary between the groups (Supplementary Fig. S2).

Figure 1d shows representative images of single cells extracted from each group to view in detail the potentially peculiar pattern of replication foci. As expected, Early, Middle, and Late S cells show recognizable patterns characterized by sparser and dimmer EdU foci located in euchromatic regions in Early, denser and brighter EdU foci with a heterochromatic disposition in Middle, large and bright EdU foci with heterochromatic disposition in Late.

We note that this analysis does not take into account the total amount of DNA in the nucleus (evaluated as the integrated intensity of the DNA marker), as this would require acquisition of z-stack across the entire volume of the nuclei^28,30^. Thus, we cannot use this information to distinguish between G1 and G2 (G2 cells have twice the DNA amount of G1 cells) or to better discriminate between Early and Late at medium pixel densities (Late cells have larger DNA amount compared to Early cells). Thus, in this work, Early and Late groups include only replication foci patterns with low pixel density, corresponding to narrower temporal windows compared to previously reported methods^28,32,33^. Conversely, the Middle subphase, as defined in our work, has a longer temporal span, including all replication foci patterns with high pixel density.

**Fig. 2:**
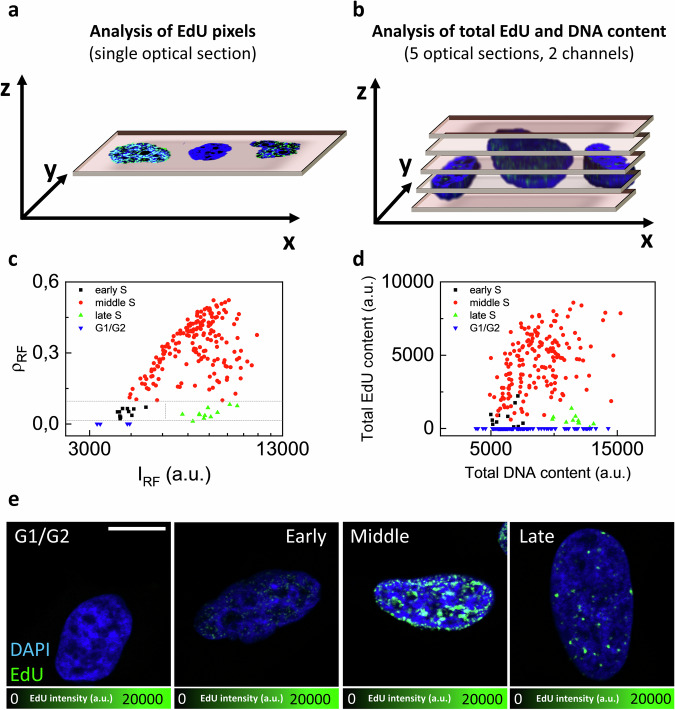
Validation of the sorting method in HeLa cells. Two different types of analysis were performed in HeLa cells to validate S-subphases classification on the basis of total EdU and DNA content. **a**, **b** Schematic comparison between (**a**) our sorting method based on the analysis of EdU pixels in a single optical section and (**b**) a standard method based on the evaluation of total EdU and DNA content from acquisition of multiple optical sections in 2 channels. For this comparison we used 5 optical sections. **c** Scatter-plot of replication foci pixel density (ρ_RF_) versus intensity (*I*_RF_) for a population of HeLa cells, as determined from a single optical section. The gray dashed lines indicate the thresholds for separation into the 4 groups: Early S-subphase (black), Middle S-subphase (red), Late S-subphase (green), and G1/G2 phases (blue). **d** Scatter plot of Total EdU content vs total DNA content obtained by the z-stack analysis, for the same cells classified in (**c**). **e** Representative confocal images of EdU replication foci in HeLa cells extracted from each group. Nuclear DNA is counterstained with DAPI, using a long incubation time (2 h). Scale bar 10 µm.

With the twofold aim of comparing our method with a widely adopted procedure and to check applicability to other cell lines, we performed an experiment in HeLa cells (Fig. 2). In this experiment, in addition to the EdU pixels analysis from a single optical section (Fig. 2a), we performed an evaluation of total EdU and DNA content from a z-stack (Fig. 2b). The scatter plot in Fig. 2c shows the replication foci pixel density vs intensity distribution of the 341 cells analyzed and the thresholds used for sorting. Representative images of single cells extracted from each group are reported in Fig. 2e, showing the characteristic patterns of replication foci associated to G1/G2 (141 cells) and Early (11 cells), Middle (176 cells), and Late (11 cells) S sub-phases. Figure 2d shows how the different sorted groups are located in a total EdU vs total DNA content scatter plot. This cross-validation analysis confirms that: (i) selected Early and Late cells are correctly located at the begin and at the end of the S phase (low vs high DNA content); (ii) we are selecting narrower temporal windows for the Early and Late S sub-phases, compared to the widely adopted procedure.

### Colocalization between replication and transcription at confocal resolution

Next, we combined the cell sorting algorithm with the Image Cross-Correlation Spectroscopy (ICCS) algorithm previously developed by our group^23,26^. As fully described in Cerutti et al. 2022, the ICCS algorithm calculates iteratively, for each cell contained in the “Count Masks”, a spatial Auto-Correlation Function (ACF) of each channel and a Cross-Correlation Function (CCF) of the two channels. The main output of the ICCS analysis is the value of colocalization fraction for each cell. The value of colocalization fraction extracted by ICCS is similar to the Pearson’s correlation coefficient^23,34^.

**Fig. 3:**
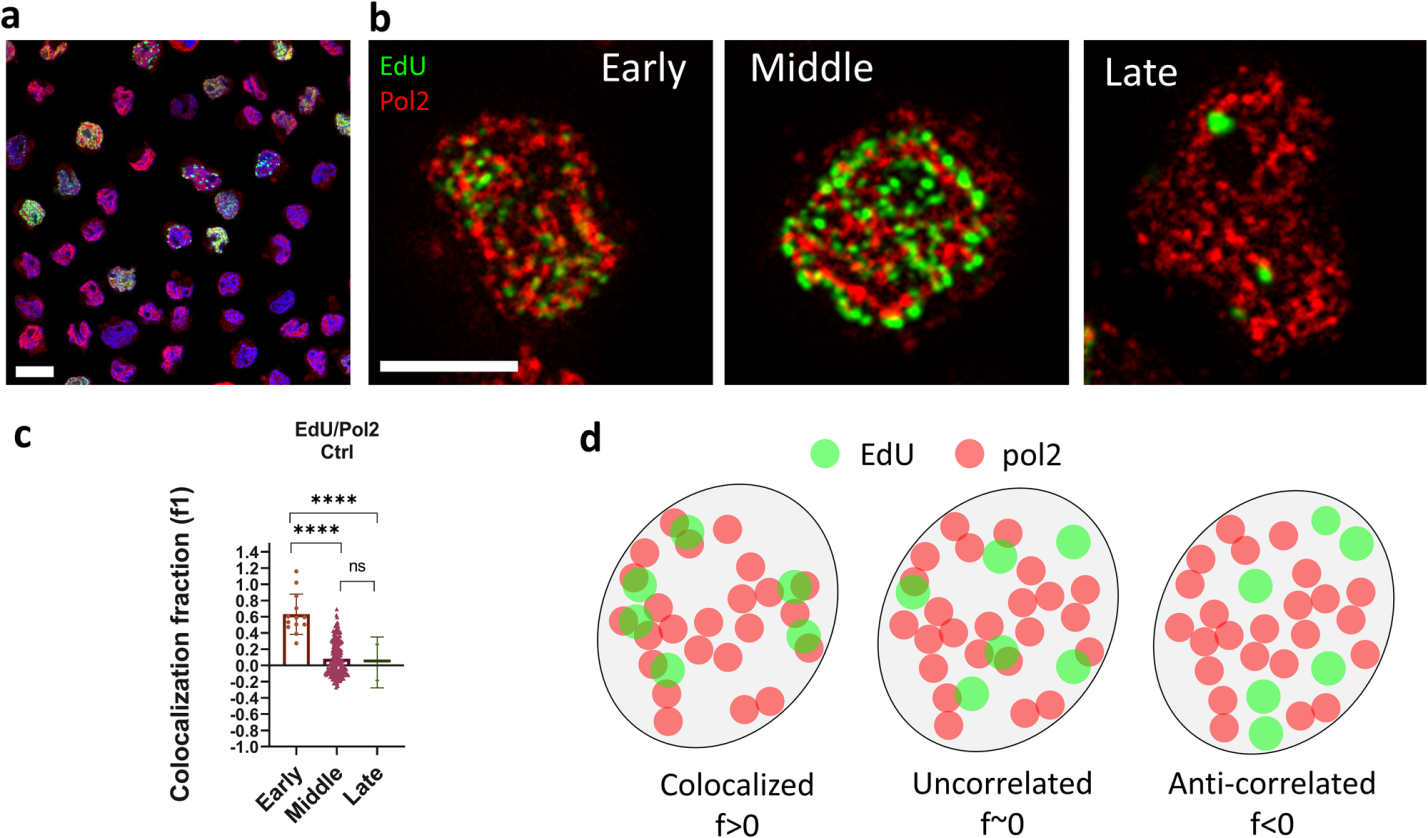
Colocalization between replication and transcription in U937-PR9 from confocal images. **a** Representative 3-color confocal image of U937-PR9 cells labeled with EdU (green), Pol2 (red), and DAPI (blue). Scale bar 10 µm. **b** Representative images of replication (EdU) and transcription (Pol2) foci in U937-PR9 cells sorted into diffferent S subphases (Early, Middle, Late). Scale bar 5 µm. **c** Colocalization fraction between replication and transcription extracted by ICCS (Early, *f*1 = 0.63 ± 0.07, mean ± s.e.m., *n* = 13 cells; Middle, *f*1 = 0.08 ± 0.01, mean ± s.e.m., *n* = 207 cells; Late, *f*1 = 0.03 ± 0.22, mean ± s.e.m., *n* = 2 cells). Mann–Whitney test was performed for statistical significance. **d** Schematic representation of the meaning of the ICCS colocalization fraction extracted from confocal images, in relation to the distribution of the replication and transcription foci.

We measured the colocalization between replication foci (EdU) and transcription foci, identified by labeling the elongating form of RNA polymerase 2 (Pol2) in U937-PR9 cells (Fig. 3a). For this analysis, we considered only cells in S phase (i.e. Early, Middle or Late). Figure 3b shows representative dual-color images of cells sorted by the algorithm in each S-subphase. The colocalization is quantified by the ICCS parameter f1, representing the fraction of signal in channel 1 (EdU) correlated with signal in channel 2. Positive values of f1 indicate some degree of colocalization, a value of 0 indicates uncorrelated particles and negative values indicate anti-correlated particles (Fig. 3d)^23^. We found that cells in the Early group have significantly higher values of colocalization (*f*1 = 0.63 ± 0.07, mean ± s.e.m., *n* = 13 cells) compared to cells in Middle (*f*1 = 0.08 ± 0.01, mean ± s.e.m., *n* = 207 cells) and Late group (Late, *f*1 = 0.03 ± 0.22, mean ± s.e.m., *n* = 2 cells) (Fig. 3c). This result indicates that, in Early group, replication foci colocalize with the region identified by the Pol2 signal, corresponding to the euchromatic domain, whilst, in Middle and Late group, this degree of colocalization is significantly lower. This result is in keeping with the time-course of the replication program: early S phase is characterized by duplication of the more euchromatic regions of the genome whereas late S phase is characterized by duplication of the more heterochromatic regions. We note that values in the Middle group are more heterogeneous as this wider group may contain duplication associated to both types of domains.

### Colocalization between replication and transcription at STED resolution

**Fig. 4:**
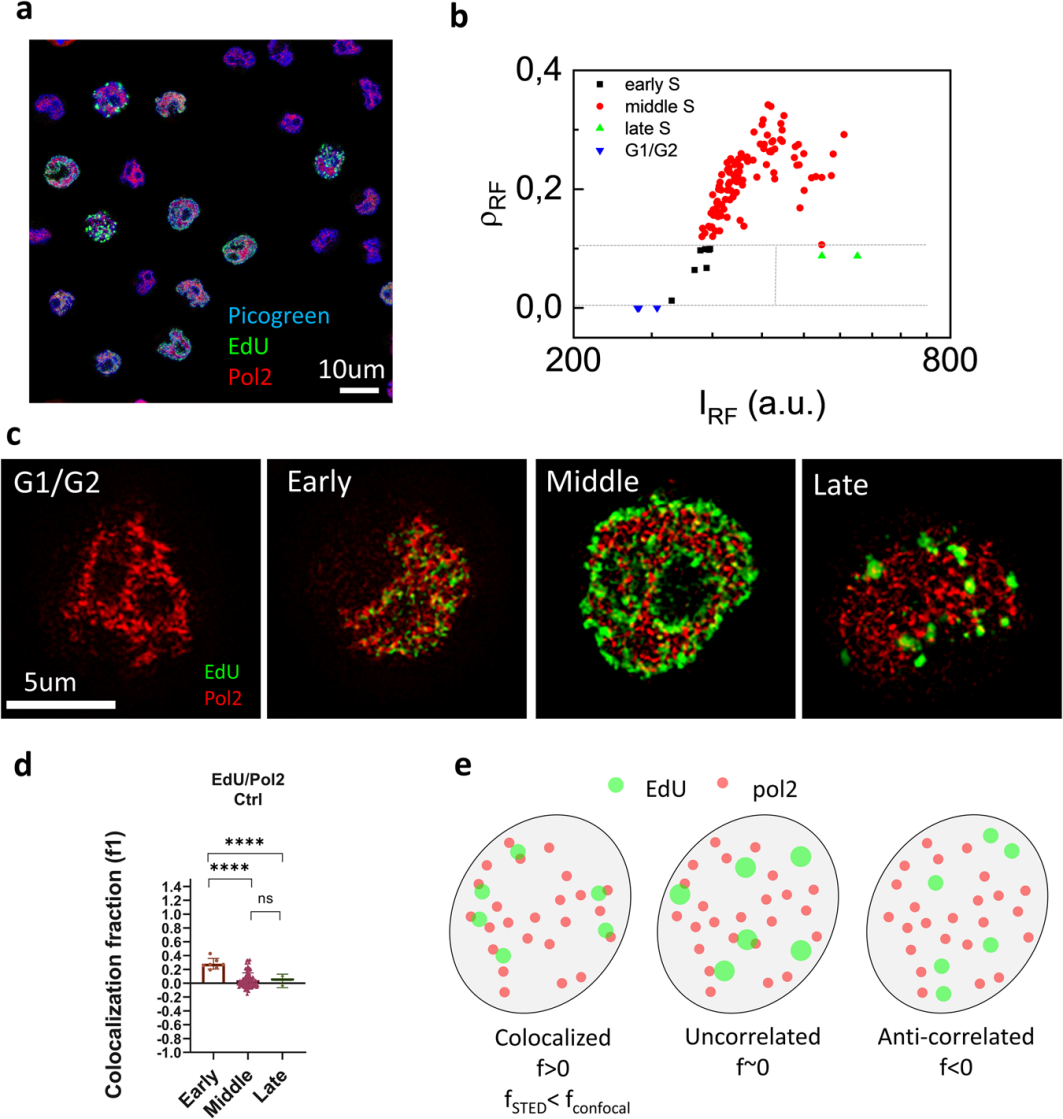
Cell cycle sorting and colocalization between replication and transcription in U937-PR9 from STED images. **a** Representative 3-color image including 2-color STED image of EdU-Alexa 594 (green) and Pol2-Atto647N (red) and confocal image of Picogreen (blue), in U937-PR9 cells. Scale bar 10 µm. **b** Scatter-plot of replication foci pixel density (ρ_RF_) versus intensity (I_RF_): the gray dashed lines indicate the thresholds for separation into the 4 groups. **c** Representative 2-color STED images of replication (EdU) and transcription (Pol2) foci in U937-PR9 cells sorted in the four cell cycle groups (G1/G2, Early, Middle, Late). Scale bar 5 µm. **d** Colocalization fraction between replication and transcription extracted by STED-ICCS. (Early, *f*1 = 0.28 ± 0.02, mean ± s.e.m., *n* = 7 cells; Middle, *f*1 = 0.04 ± 0.01, mean ± s.e.m., *n* = 110 cells; Late, *f*1 = 0.03 ± 0.06, mean ± s.e.m., *n* = 2 cells). Mann–Whitney test was performed for statistical significance. **e** Schematic representation of the meaning of the ICCS colocalization fraction extracted from STED images, in relation to the distribution of the replication and transcription foci.

Next, we applied the sorting and ICCS algorithm to images of U937-PR9 cells acquired by Stimulated emission depletion (STED) microscopy. STED is a super-resolution microscopy technique with a huge potential to investigate sub-cellular structures that cannot be accurately resolved by light microscopy. STED microscopy has in common many features with confocal microscopy, such as optical sectioning, depth penetration and imaging speed^14^. Specifically, we used Tau-STED microscopy^35^, a commercially available implementation of a method called separation of photon by lifetime tuning (SPLIT)^36–38^. In this method, the improvement of spatial resolution and the subtraction of background are simultaneously provided by the phasor analysis of lifetime^36^. We had previously determined that Tau-STED microscopy provided better performances in U937-PR9 cells compared to conventional STED microscopy^23^. In this case, DNA was stained with Picogreen, EdU was labeled with Alexa 594 azide, Pol2 with Atto 647 N, the STED wavelength being 775 nm (Fig. 4a).

The analysis included 12 images with an image size of approximately 100 × 100 μm. Figure 4b shows the replication foci pixel density vs intensity distribution of the 245 cells analyzed. Also, in this case, all cells showing no EdU signal (number of pixels below 10) were classified as G1/G2 (126 cells). All cells with a density above the threshold ρ_thr_ were classified as Middle (110 cells). Cells with a density below the threshold ρ_thr_ and intensity below a threshold I_thr_ were classified as Early (7 cells). Cells with a density below the threshold ρ_thr_ and intensity above the threshold I_thr_ were classified as Late (2 cells). Figure 3c shows representative dual-color images of cells sorted by the algorithm in the 4 groups.

We found that cells in the Early group have significantly higher values of colocalization (*f*1 = 0.28 ± 0.02, mean ± s.e.m., *n* = 7 cells) compared to cells in Middle (*f*1 = 0.04 ± 0.01, mean ± s.e.m., *n* = 110 cells) and Late group (*f*1 = 0.03 ± 0.06, mean ± s.e.m., *n* = 2 cells) (Fig. 4c). Thus, the STED results confirm that, in Early group, replication foci colocalize with the region identified by the Pol2 signal, corresponding to the euchromatic domain, whilst, in Middle and Late group, this degree of colocalization is significantly lower.

The values of colocalization of the Early group are lower in STED (*f*1 = 0.28) compared to confocal (*f*1 = 0.63). This can be explained by the higher spatial resolution provided by lifetime-based STED microscopy (~60 nm)^39^ compared to confocal microscopy (*R* ~200 nm). In the STED images, the foci appear as smaller green/red spots (Fig. 4e) which overlap less in comparison to the foci in the confocal images (Fig. 3d), resulting in an overall lower degree of cross-correlation. Thus, the value of f1 provided by STED represents a quantitative measure of the colocalization between replication and transcription at a smaller spatial scale.

### Effects of the PML-RARα oncogene on the colocalization between replication and transcription

Activation of the PML-RARα oncogene induces disruption of PML bodies and the appearance of PML-RARα speckles in the nucleus of U937-PR9 cells^23^ (Supplementary Fig. S3). The expression of the oncoprotein may induce alterations in the organization of basic genomic processes, including the progress of replication during the S phase and its coordination with transcription. For this reason, we monitored if the colocalization between replication and transcription was altered following activation of the oncogene PML-RARα in U937-PR9 cells.

**Fig. 5:**
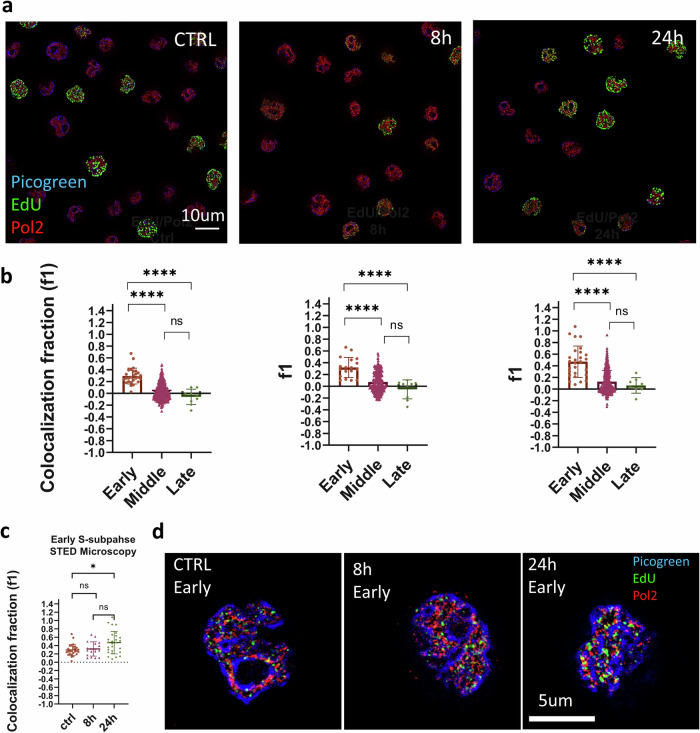
Effects of the PML-RARα oncogene on the colocalization between replication and transcription. **a** Representative images of U937-PR9 cells before (CTRL) and after PML-RARα oncogene activation (8 h, 24 h). The image includes a 2-color STED image of EdU-Alexa 594 (green) and Pol2-Atto647N (red). Nuclear DNA is counterstained with Picogreen. Scale bar 10 µm. **b** Colocalization fraction between replication and transcription extracted by STED-ICCS for the control (left) and activated samples. (center and right). Cumulative results for control cells Early, *f*1 = 0.30 ± 0.02, mean ± s.e.m., *n* = 27 cells; Middle, *f*1 = 0.02 ± 0.007, mean ± s.e.m., *n* = 343 cells; Late, *f*1 = −0.05 ± 0.04, mean ± s.e.m., *n* = 9 cells) (Left). Results for cells at 8 h after PML-RARα activation (Early, *f*1 = 0.32 ± 0.04, mean ± s.e.m., *n* = 18 cells; Middle, *f*1 = 0.07 ± 0.009, mean ± s.e.m., *n* = 286 cells; Late, *f*1 = −0.05 ± 0.05, mean ± s.e.m., *n* = 9 cells) (Center). Results for cells at 24 h after PML-RARα activation (Early, *f*1 = 0.47 ± 0.05, mean ± s.e.m., *n* = 27 cells; Middle, *f*1 = 0.12 ± 0.011, mean ± s.e.m., *n* = 302 cells; Late, *f*1 = 0.06 ± 0.04, mean ± s.e.m., *n* = 8 cells) (Right). Mann–Whitney test was performed for statistical significance. **c** Colocalization fraction between replication and transcription in Early S phase before and after PML-RARα oncogene activation (CTRL, *f*1 = 0.30 ± 0.02, mean ± s.e.m., *n* = 27 cells; 8 h, *f*1 = 0.31 ± 0.04, mean ± s.e.m., *n* = 18 cells; 24 h, *f*1 = 0.47 ± 0.05, mean ± s.e.m., *n* = 27 cells). Mann–Whitney test was performed for statistical significance (24 h vs control, *p* = 0.012). **d** Representative 2-color STED images of replication (EdU) and transcription (Pol2) foci in U937-PR9 cells in Early S phase before (CTRL Early) and after PML-RARα oncogene activation (8 h Early, 24 h Early). Scale bar 5 µm.

To this aim, we treated the cells with 0.1 mM ZnSO_4_ and performed STED imaging at two different time points (8 h and 24 h) (Fig. 5a). We then applied the sorting algorithm to separate cells in different S subphases (Supplementary Fig. S4) and measured the colocalization between replication (EdU) and transcription (Pol2) by ICCS. For the 8 h and 24 h samples, we found that the colocalization parameter f1 was higher in the Early group compared to the Middle and Late group, similarly to control cells (Fig. 5b). This indicates that, both before and after activation of the oncogene, the replication foci classified as Early are located in the euchromatic domain of the nucleus whereas the replication foci classified as Middle or Late are in the heterochromatic domain of the nucleus. Thus, these very general features of the replication program are maintained after oncogene activation.

Next, we focused our attention on the Early group and compared the ICCS colocalization parameter f1 before and after activation of the oncogene (Fig. 5c). We found that the colocalization between replication and transcription was significantly higher in the 24 h sample than in the control sample (control: *f*1 = 0.30 ± 0.02, mean ± s.e.m., *n* = 27 cells; 24 h: *f*1 = 0.47 ± 0.05, mean ± s.e.m., *n* = 27 cells; 24 h vs control, *p* = 0.012). The observed increase of the colocalization parameter indicates a higher level of proximity between replication and transcription foci, in the Early phase, following activation of the oncogene.

## Discussion

Spatio-temporal organization of the genome during cell cycle progression is crucial for the control of cell proliferation and oncogenesis. So far, not much attention has been paid to the intimate role of S-phases in cancer and, above all, which cell cycle phase is mainly involved during oncogene activation. In this work, we propose a semi-automatic image-based approach to sort the cells into 4 cell cycle subphases (namely Early S, Middle S, Late S, G1/G2), and analyze them with image cross-correlation spectroscopy (ICCS). The ICCS method was well described previously in relation to confocal^25^ and super-resolution^26,40,41^ microscopy. In particular, ICCS facilitates colocalization analysis by avoiding pre-segmentation of the image into objects. Recently, it was improved by Cerutti et al. by introducing the automatic analysis of a large number of cells, identified using a “Count mask” generated on ImageJ^23^. Here, we show that we can automatically analyze a large number of cells by ICCS and also sort them according to the progression along the cell cycle. Compared to standard methods based on the evaluation of the total content of DNA and EdU from 3D stacks^28,30^, the sorting in our method is based only on the evaluation of the number and the intensity of pixels in a single optical section of the replication foci image. Thus, the method can be useful in all those cases in which an accurate evaluation of total DNA content is not available. We found that our selection of Early and Late correspond to shorter temporal windows at the begin and at the end of the S phase (replication foci patterns with low pixel density), respectively, whereas our selection of the Middle subphase has a wider temporal span (replication foci patterns with high pixel density). As a case study, we use ICCS to evaluate the spatial coordination between two basic cell mechanisms such as DNA replication and DNA transcription, at different sub-phases of the S phase, in U937-PR9, a model of PML-RARα oncogene activation.

STED and Confocal microscopes are two potent imaging technologies whose potential is extremely useful in single cell quantitative analysis performed in this project. The results obtained in this work show that Early-S-Subphase is the cell cycle phase when DNA replication and DNA transcription are more spatially cross-correlated. This result is in keeping with the organization of the replication program: euchromatic regions of the genome (regions which are also highly transcribed) are duplicated in the early S phase whereas the heterochromatic regions (regions which are not transcribed) are duplicated in the middle and late S phases. This result was confirmed in both microscopy technologies (see Figs. 3c, 4d). Nevertheless, the value of STED colocalization of the Early group was lower compared to confocal colocalization of the same group, due to the higher resolution of the STED images.

Thanks to this new tool, we monitored if the colocalization between replication and transcription was altered following activation of the oncogene PML-RARα in U937-PR9 cells. We found that, also after activation of the expression of PML-RARα, colocalization between replication and transcription was higher in the Early phase compared to the Middle and Late phase, similarly to what we found for control cells. Thus, both before and after activation of the oncogene, replication foci are located in the euchromatic domain of the nucleus during the Early S phase while they are in the heterochromatic domain of the nucleus during the Middle and Late S phase. Interestingly, we found that the colocalization between replication and transcription in the Early S phase was higher 24 h after PML-RARα activation when compared to the control sample. This study was limited in the number of colors that we could detect simultaneously, so we could not visualize, on the same cell, the distribution of the PML-RARα oncoprotein or markers of DNA damage, to determine if the observed increase of proximity was correlated to a higher expression of the oncoprotein and/or to an increase of DNA damage formation. Another limitation was the total number of cells analyzed which was about an order of magnitude smaller compared to reported quantitative image cytometry methods based on widefield microscopy^42^. In general, higher spatial resolution is associated with smaller fields of view and a lower number of cells that can be acquired in each amount of time. Nevertheless, we believe that our approach could also be used with images acquired at lower spatial resolution and with larger field of view, containing a proportionally higher number of cells (Supplementary Figs. S5 and S6).

Oncogenes may promote genomic instability through different mechanisms, including replication and transcription stress^43^. Our imaging-based method suggests that the PML-RARα oncogene induces global alterations in the spatio-temporal coordination between replication and transcription during the Early S phase, detected as an average increase of the proximity between the replication and transcription processes. Future efforts will be aimed at determining if this average increase in proximity is linked to global chromatin alterations, e.g. an average increase of chromatin compaction^44,45^, or to more localized effects, e.g. an increase in the rate of transcription-replication collisions^46^.

## Methods

### Cell culture and treatments

U937-PR9 cells were grown in RPMI-1640 medium (Sigma-Aldrich R7388) with the addition of 10% fetal bovine serum (Sigma-Aldrich F9665) and 1% penicillin/streptomycin (Sigma-Aldrich P4333) and maintained at 37 °C and 5% CO_2_. To induce the expression of PML-RARα, the cells were incubated with 0.1 mM ZnSO_4_ solution and left growing for 8 h or 24 h. HeLa cells were grown in Dulbecco’s Modified Eagle Medium (DMEM), high glucose (Gibco 11965092) supplemented with 10% Fetal bovine serum (Sigma-Aldrich F9665) and 1% penicillin-streptomycin (Sigma-Aldrich P4333) and maintained at 37 °C and 5% CO_2_. To label replication foci, cells were incubated with the thymidine analog 5-Ethynyl-2′-deoxyuridine (EdU) (Thermo Fisher Scientific) at 10 μM for 25 min at 37 °C and 5% CO_2_. The cells were seeded on poly-L-lysine (Sigma-Aldrich P8920) coated glass coverslips immediately before the experiments.

### Fluorescence labeling

Cells were fixed with 4% paraformaldehyde (w/v) for 10 min at room temperature, and permeabilized with 0.5% (v/v) Triton X-100 in Phosphate Buffer Saline (PBS) for 20 min. To label the incorporated EdU, cells were then incubated for 30 min with the Click-iT reaction cocktail containing Alexa Fluor 488 azide (Invitrogen C10337) or Alexa Fluor 594 azide (Invitrogen C10639), according to the manufacturer’s instructions. Cells were then blocked with 3% BSA in PBS and incubated in a wet chamber with primary antibody opportunely diluted in Incubation Buffer, overnight at 4 °C. Cells were then extensively washed with Washing Buffer (WB) 3 × 15 min and incubated with secondary antibody diluted in Incubation Buffer for 1 h at room temperature, followed by the same washing procedure with WB. Finally, cells were extensively washed with PBS, incubated with DNA dyes dilutions for 10 min, and then mounted on glass slides with ProLong Diamond Antifade Mountant (Invitrogen P36961). Only for the experiment with HeLa cells, in which we evaluated the total DNA content, the DAPI incubation time was 2 h, to guarantee complete labeling of the nuclear DNA.

Primary antibodies used in this work are RNA polymerase II CTD repeat YSPTSPS (phospho S2) rabbit (ab5095) (EPR18855) (ab193468) (hereinafter referred to as Pol2), and PML mouse (sc-966, Santa Cruz Biotechnology). Secondary antibodies used in this work are goat α-Mouse IgG H + L Alexa Fluor 488 (ab150113, abcam), goat α-Rabbit Atto 594 (77671 Sigma-Aldrich) and goat α-Rabbit Atto 647 N (40839 Sigma-Aldrich). DNA dyes used in this study are DAPI (62248 Thermo Fisher Scientific, Waltham, MA, USA) and Picogreen (P7581 Sigma-Aldrich).

### Image acquisition

Confocal image acquisition was performed using a Leica TCS SP8 confocal microscope. An HCX PL APO CS2 63 × 1.40 NA oil immersion objective lens (Leica Microsystems, Mannheim, Germany) was used. Excitation wavelengths/emission bandwidths were the following: DAPI (405/410–483), Alexa 488 (488/500–550), and ATTO 594 (561/589–643). The pinhole size was set to 0.8 Airy Units at a wavelength of 580 nm. Images were acquired with 2048 × 2048 pixels and 45 nm of pixel size.

For the experiment with HeLa cells, images were acquired as z-stacks made of 5 optical sections with z-step of 1 µm. Images were made by 2048 × 2048 pixels with a pixel size of 70 nm.

For the data shown in Supplementary Fig. S5, images were acquired using a HC PL APO CS2 40 × 1.3 NA objective with 2048 × 2048 pixels and 71 nm of pixel size. For the data shown in Supplementary Fig. S6, images were acquired using a HC PL APO CS2 20 × 0.75 NA objective with 2048 × 2048 pixels and 142 nm of pixel size.

STED image acquisition was performed on a Leica Stellaris 8 Tau-STED microscope, using an HC PL APO CS2 100x/1.40 oil immersion objective lens (Leica Microsystems, Mannheim, Germany). Emission depletion was accomplished with a 775 nm STED laser. A white light laser provided excitation at the desired wavelength for each sample. The setting of excitation wavelengths/emission bandwidths were the following: Picogreen (488/ 500–550), Alexa 594 (590/ 595-641), Atto647N (646, 651–720). Images were acquired with 2048 × 2048 pixels and a pixel size of 45 nm. Additional parameters were Tau-Strength at 100, denoise at 50 and background suppression checked.

### Image pre-processing

The acquired images were pre-processed on Fiji^47^ to obtain the suitable input files to run the used algorithms for Cell Cycle sorting and Image Cross-Correlation Spectroscopy (ICCS) well explained in subsequent paragraphs.

The “count masks” (nuclei selection masks) were generated as follows (Supplementary Fig. S7a): the images of the DNA channel were converted into binary images using the function “threshold” of ImageJ, using the “Default” threshold algorithm; the nuclei were identified and listed as objects using the “analyze particles” function and the images of the “count masks” were saved. Cells in mitosis were not included in the analysis.

The replication foci binary images were obtained from the replication foci images using the function “threshold” of ImageJ, using the “Default” threshold algorithm, to exclude background pixels from the analysis (Supplementary Fig. S7b). The value of the intensity threshold was set at a value ~ 1.5x the value of the background level, estimated from the EdU intensity of G1/G2 cells in the first frame of the dataset (Fig. S7b).

The intensity threshold value was set at the same value when analyzing images acquired in the same experiment.

The background was subtracted from the intensity images using the function “Subtraction of Background” (Rolling ball of 10 pixels).

### Algorithm for sorting the cells based on the cell cycle phase

The sorting algorithm was implemented as a custom script in Matlab. The script requires (i) a stack containing the “count masks”; (ii) a stack containing the replication foci images; (iii) a stack containing the binary images obtained after thresholding the replication foci images. For each nucleus j of the count mask, the algorithm calculates the parameters *ρ*_RF_ (replication foci pixel density) and *I*_RF_ (replication foci pixel intensity), defined as:

1 $${{{\rm{\rho }}}}{{{\rm{RF}}}}({{{\rm{j}}}})={N}_{{{{\rm{RF}}}}}/{N}_{{{{\rm{nuc}}}}}$$

2 $${I}_{{{{\rm{RF}}}}}({{{\rm{j}}}})={ < I({{{\rm{x}}}},{{{\rm{y}}}}) > }_{{{{\rm{j}}}}}$$

Where *N*_RF_ is the number of pixels in the replication foci binary image, *N*_nuc_ is the number of pixels in the nucleus, *I*(x,y) is the replication foci image, and the brackets indicate averaging over the replication foci pixels.

The values of density *ρ*_RF_ and intensity *I*_RF_ from all the cells in the count masks are used to generate a scatter plot and to determine the sorting criteria.

Cells are classified as G1/G2 if *ρ*_RF_(j)<*ρ*_min_, with *ρ*_min _= *N*_min_/<*N*_nuc_>, where *N*_min_ is the minimum number of pixels to consider a cell as containing replication foci and <*N*_nuc_> is the average value of nuclear area in the population of cells, in pixels.

Cells are classified as Middle if *ρ*_RF_(j) > *ρ*_thr_, with *ρ*_thr_ = *k*_ρ_ *ρ*_max_, where *ρ*_max_ is the maximum value of *ρ*_RF_(j) in the population of cells and *k*_ρ_ is a multiplying factor. Cells are classified as Early or Late if *ρ*_min_ < *ρ*_RF_(j)<*ρ*_thr_. In this subgroup, cells are classified as Early if *I*_RF_(j) < *I*_thr_, with *I*_thr_ = *k* *I*_min_, where I_min_ is the minimum value of *I*_RF_(j) in the Early or Late subgroup and k is a multiplying factor. Cells are classified as Late if *I*_RF_(j) > *I*_thr_. Finally, we exclude from the Early group the cells with a size of the nucleus larger than a given threshold, namely if *N*_nuc_(j) > *k*_size_<*N*_nuc_>.

All the cell cycle phase selection thresholds (*N*_min_, *k*_ρ_, *k*, *k*_size_) were initialized to the following default values: *N*_min_ = 10 pixels, *k*_ρ_ = 0.4, *k* = 3, *k*_size_ = 1.2. When necessary (for instance for the experiments with the HeLa cell line), these values were adjusted by looking at the scatter plot (Fig. S1) and at the images of cells at the boundaries between the Early S group and the other groups (Fig. S2). In all cases, the selection thresholds were set at the same values when analyzing images acquired in the same experimental conditions, to avoid any bias in the results.

### Image Cross-Correlation Spectroscopy analysis

The Image Cross-Correlation Spectroscopy (ICCS) analysis was based on a modified version of the ICCS algorithm^26^ (https://github.com/llanzano/ICCS), well described in ref. ^23^. The algorithm was performed in MATLAB (The MathWorks, Natick, Massachusetts). The advantage of the modified version of ICCS is the automatic calculation of ICCS parameters on single cells starting from multiple input image files containing many cells. The main algorithm output is the parameter *f*_1_ (*f*_2_) values which represent the fraction of signal in channel 1 (channel 2) which is cross-correlated with the other channel. Values of this parameter range from 1 (maximum cross-correlation), to 0 (no cross-correlation), to −1 (maximum anti-correlation)^23^.

### Statistics and reproducibility

Statistics analyses were performed using GraphPad Prism version 8.0.0 for Windows, GraphPad Software, San Diego, California USA, www.graphpad.com. Mann–Whitney test was performed group by group and assumed a non-Gaussian distribution and an unpaired experimental design. The Mann–Whitney test compares the median of the two groups to find the difference between the distribution of ranks among the two groups.

### Reporting summary

Further information on research design is available in the Nature Portfolio Reporting Summary linked to this article.

## Supplementary information

SUPPLEMENTAL MATERIAL

Description of Additional Supplementary Files

Supplementary Data 1

Reporting Summary

## Data Availability

The source data behind the graphs in the paper are available in Supplementary Data 1. All other data are available from the corresponding author upon reasonable request.

## Code availability

The custom MATLAB code used in this study is available at https://github.com/llanzano/cellcycle^48^.
