# Supplementary material for: Super-resolved analysis of colocalization between replication and transcription along the cell cycle in a model of oncogene activation
Source: Commun Biol. 2024 Oct 4;7:1260. doi: 10.1038/s42003-024-06972-2 (PMC11452374; doi:10.1038/s42003-024-06972-2)
Supplement: Supplementary file 1 — SUPPLEMENTAL MATERIAL [file 42003_2024_6972_MOESM1_ESM.pdf]

Supporting Information for

Super-resolved analysis of colocalization between replication and transcription along the cell cycle in a model of oncogene activation

Anna Provvidenza Privitera[1#], Silvia Scalisi[1#], Greta Paternò[1], Elena Cerutti[1,2], Morgana D'Amico[1], Pier Giuseppe Pelicci[3,4], Mario Faretta[3], Gaetano Ivan Dellino[3,4,*], Alberto Diaspro[2,5], Luca Lanzanò[1,2,*]

[1]Department of Physics and Astronomy "Ettore Majorana", University of Catania, Via S. Sofia 64, 95123 Catania, Italy
[2]Nanoscopy and NIC@IIT, CHT Erzelli, Istituto Italiano di Tecnologia, Via Enrico Melen 83, Building B, 16152 Genoa, Italy
[3]Department of Experimental Oncology, IEO, European Institute of Oncology IRCCS, 20100 Milan, Italy
[4]Department of Oncology and Hemato-Oncology, University of Milan, 20100 Milan, Italy
[5]DIFILAB, Department of Physics, University of Genoa, via Dodecaneso 33, 16143 Genoa, Italy

*Corresponding authors: luca.lanzano@unict.it; gaetano.dellino@ieo.it

[#]Equally contributing authors

The file contains:

Supplementary Figures S1-S7

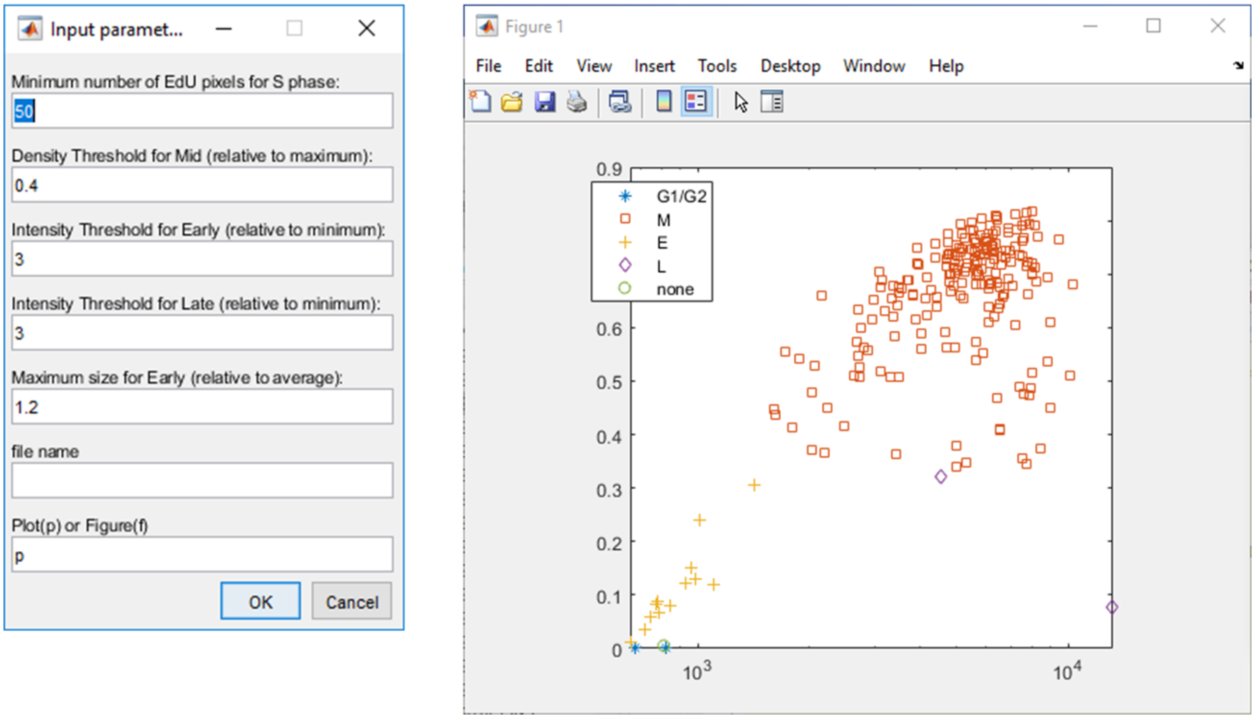

Fig.S1 Selection of the threshold values with scatter plot.

Screenshot of the Matlab script input menu with the option of visualizing the scatter plot (replication foci pixel density vs intensity). The threshold values are selected, and the scatter plot shows the distribution of the sorted cells in the scatter plot.

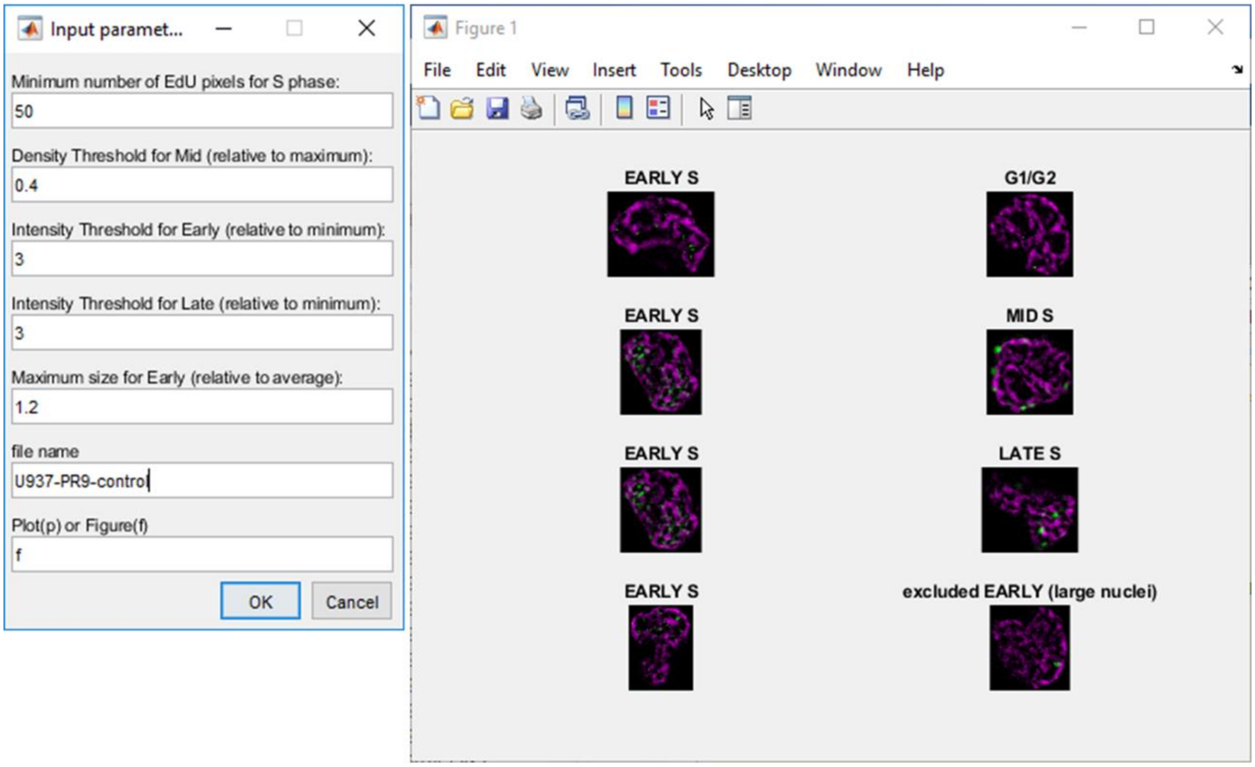

Fig.S2 Selection of the threshold values with representative images.

Screenshot of the Matlab script input menu with the option of visualizing representative images of cells at the boundaries of the Early S group with the other groups. The threshold values are selected and the images are updated.

First row: cell of the Early S group with minimum density of pixels and cell of the G1/G2 group with maximum density of pixels. Second row: cell of the Early S group with maximum density of pixels and cell of the Middle S group with minimum density of pixels. Third row: cell of the Early S group with maximum pixel intensity and cell of the Late group with minimum pixel intensity. Fourth row: cell of the Early S group with maximum nuclear size and cell of the excluded cells group with minimum nuclear size.

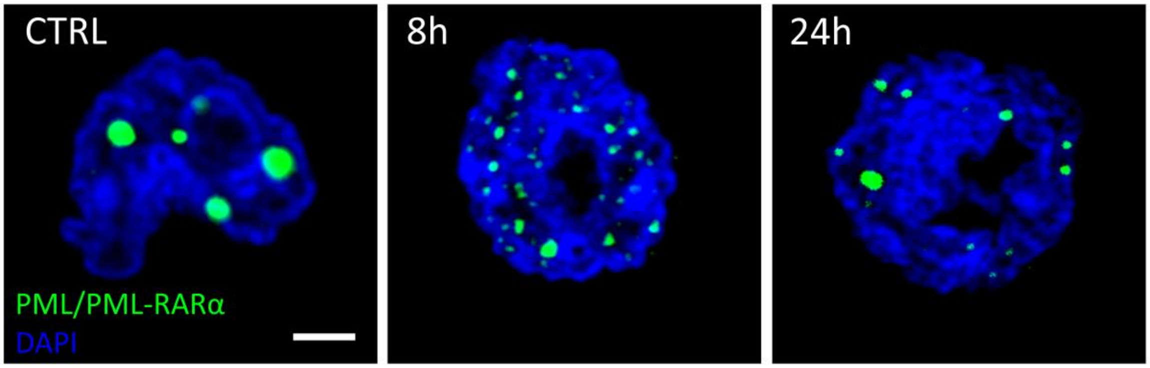

Fig.S3 Activation of the PML-RARα oncogene in the U937-PR9 cell line.

Representative 2-color confocal images of U937-PR9 cells labeled with PML (green) and DAPI (blue), under basal conditions (CTRL), and treated with a solution of $ZnSO_4$ 0.1 mM for 8 h, and for 24 h to activate the expression of PML-RARα oncoprotein. Scale bar 2 µm.

Experiment 1

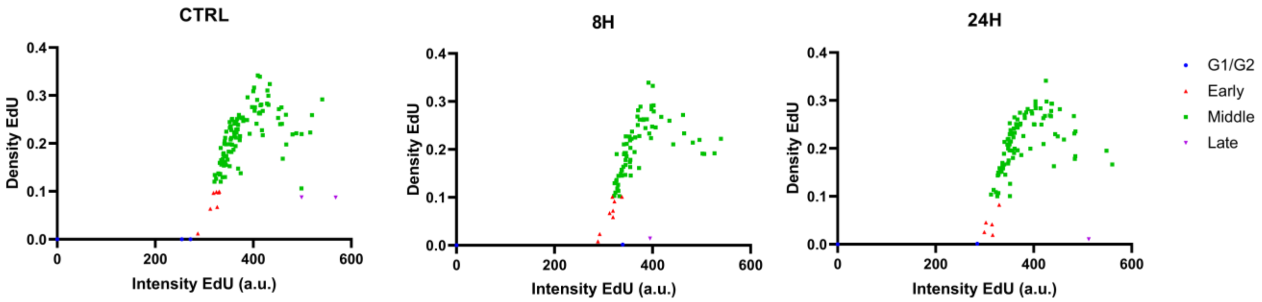

Experiment 2

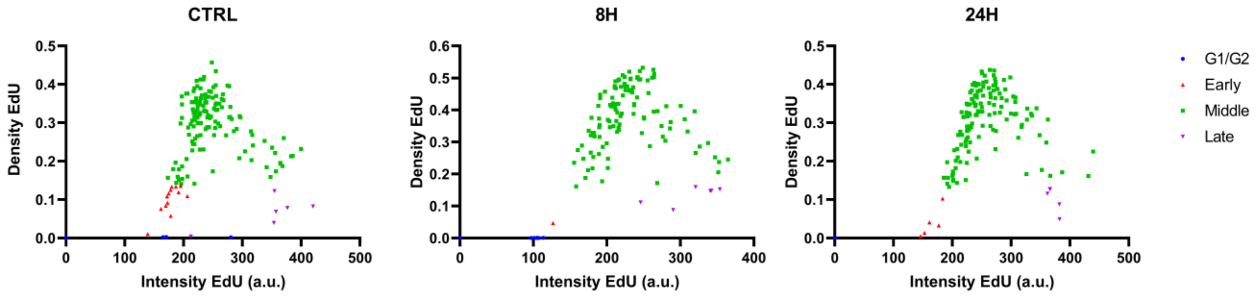

Experiment 3

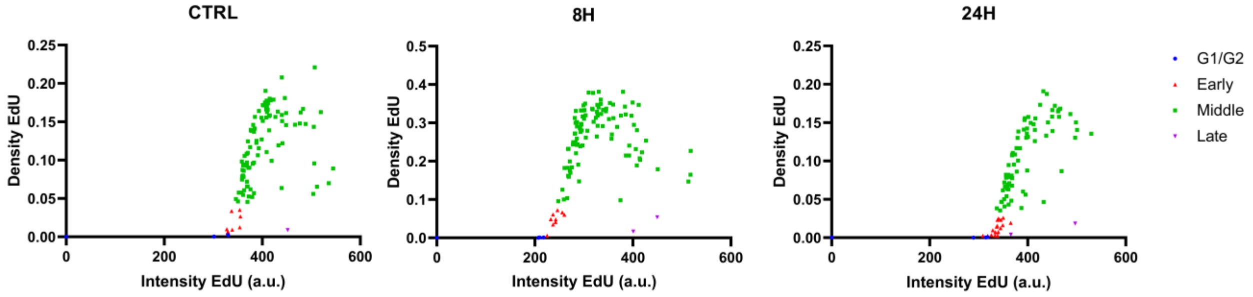

Fig.S4 Scatter Plots for the different experiments involving PML-RARα oncogene activation.

Scatter-plot of replication foci pixel density (ρ_{RF}) versus intensity (I_{RF}) for the 3 experiments related to PML-RARα oncogene activation. In each experiment, we analyzed cells under basal conditions (CTRL), and treated with a solution of $ZnSO_4$ 0.1 mM for 8 h, and for 24 h to activate the expression of PML-RARα oncoprotein.

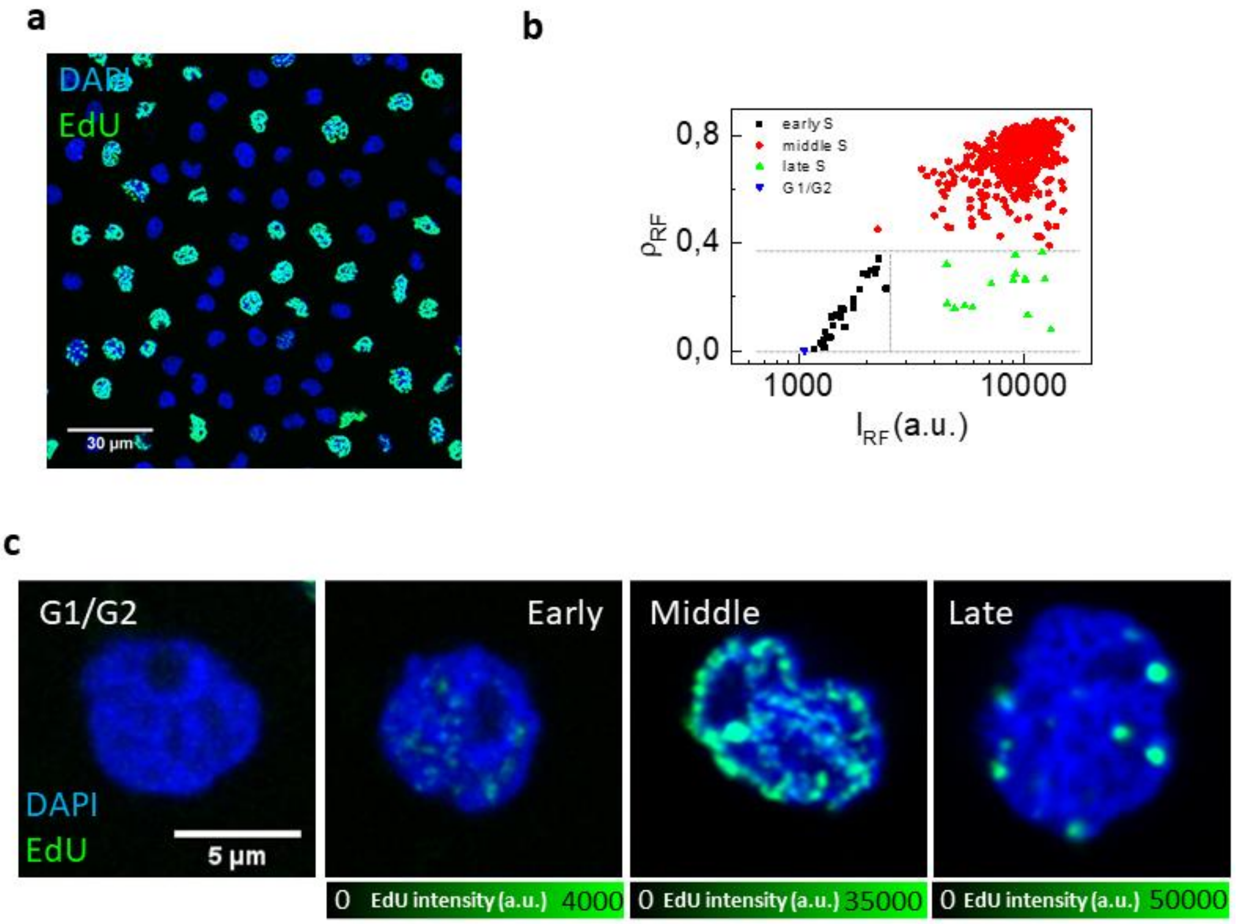

Fig.S5. Application of the algorithm to images acquired with a 40x objective.

a) Representative confocal image of U937-PR9 cells labelled with DAPI (blue) and EdU-Alexa 488 (green), acquired using a 40x objective. b) Scatter-plot of replication foci pixel density (ρ_{RF}) versus intensity (I_{RF}): the grey dashed lines indicate the thresholds for separation into the 4 groups. Each data point represents a single cell of a total of 981 cells. Early S-subphase (black), Middle S-subphase (red), Late S-subphase (green) and G1/G2 phases (blue). G1/G2 cells with I_{RF}=0 are not visible in the plot. c) Representative images of EdU replication foci in U937-PR9 cells extracted from each group.

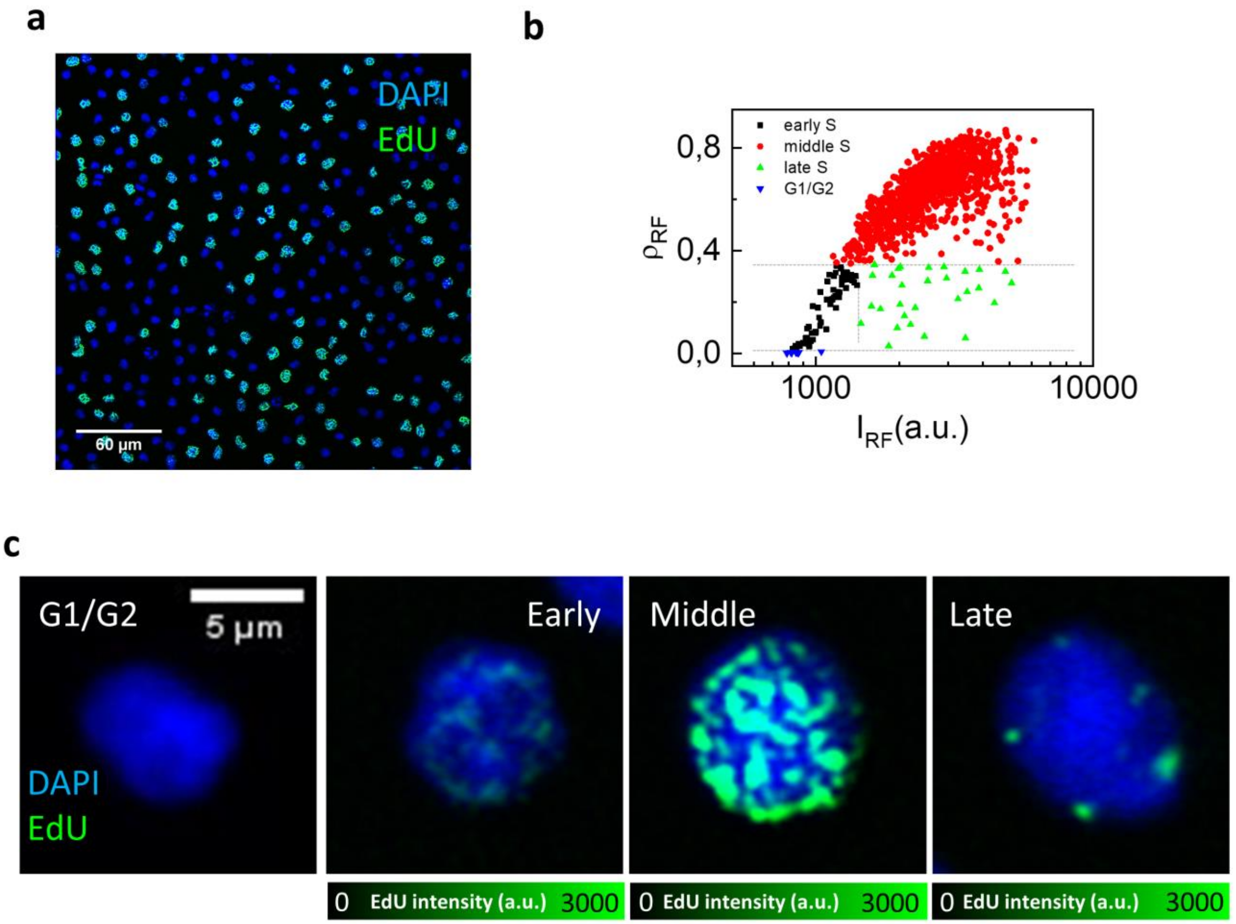

Fig.S6. Application of the algorithm to images acquired with a 20x objective.

a) Representative confocal image of U937-PR9 cells labelled with DAPI (blue) and EdU-Alexa 488 (green), acquired using a 20x objective. b) Scatter-plot of replication foci pixel density (ρ_{RF}) versus intensity (I_{RF}): the grey dashed lines indicate the thresholds for separation into the 4 groups. Each data point represents a single cell of a total of 2531 cells. Early S-subphase (black), Middle S-subphase (red), Late S-subphase (green) and G1/G2 phases (blue). G1/G2 cells with I_{RF}=0 are not visible in the plot. c) Representative images of EdU replication foci in U937-PR9 cells extracted from each group.

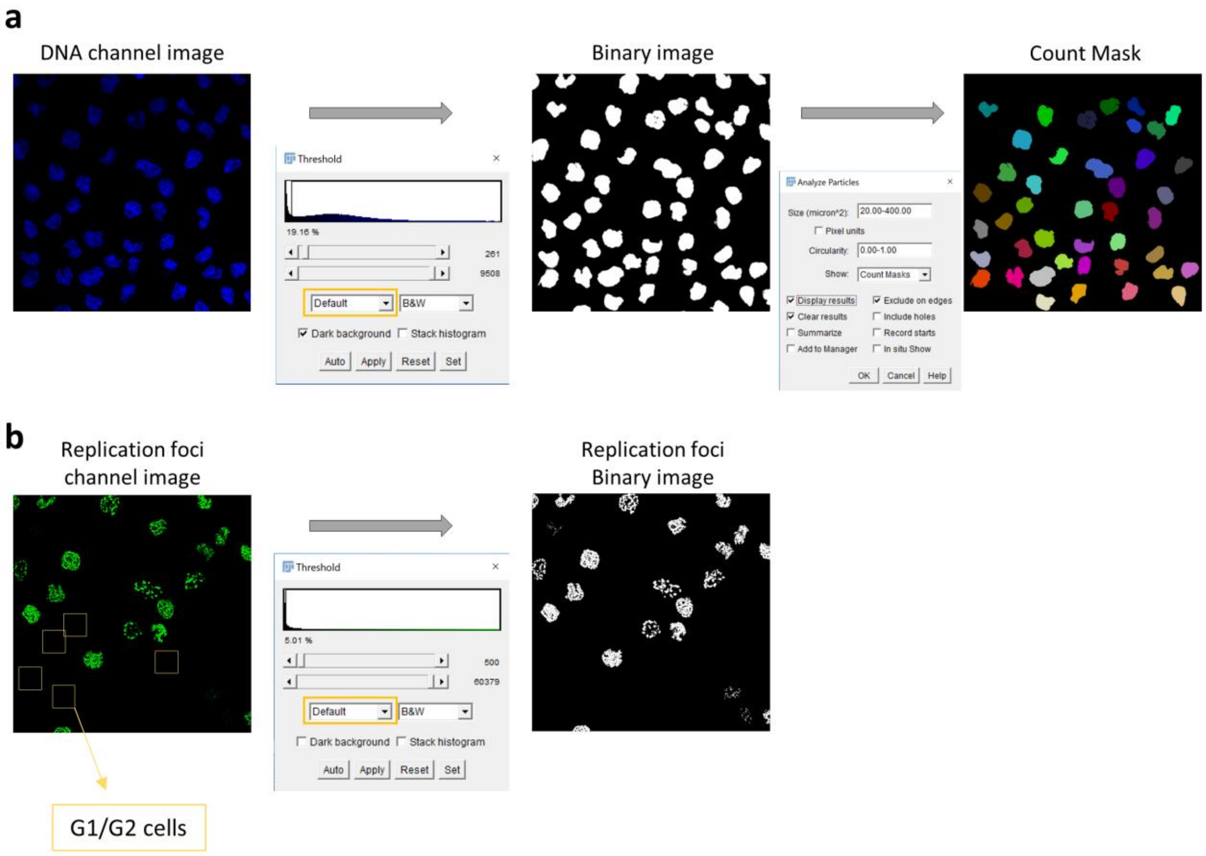

Fig.S7. Generation of the Count Masks of the nuclei and the replication foci binary images.

(a) Each image of the DNA channel is converted into a binary image using the Threshold function of ImageJ (Default method), then a Count Mask is generated using the Analyze Particles function of ImageJ. (b) Each image of the replication foci is converted into a binary image using the Threshold function of ImageJ (Default method). The threshold value is set at a value equal to ~1.5x the maximum background intensity detected in G1/G2 cells.
